# Increased cortisol metabolism in women with pregnancy-related hypertension

**DOI:** 10.1007/s12020-018-1586-4

**Published:** 2018-04-02

**Authors:** Katarzyna Kosicka, Anna Siemiątkowska, Agata Szpera-Goździewicz, Mariola Krzyścin, Grzegorz H. Bręborowicz, Franciszek K. Główka

**Affiliations:** 1 Department of Physical Pharmacy and Pharmacokinetics, Poznan University of Medical Sciences, 6 Święcickiego Street, Poznań, 60-781 Poland; 2 Department of Perinatology and Gynecology, Poznan University of Medical Sciences, 33 Polna Street, Poznań, 60-535 Poland

**Keywords:** Cortisol, Tetrahydrometabolites, 11β-hydroxysteroid dehydrogenase, 5β-reductase

## Abstract

**Purpose:**

The diminished function of 11β-hydroxysteroid dehydrogenase 2 (11β-HSD2) was found in placentae from preeclamptic pregnancies. Here, we examine the overall maternal glucocorticoid balance in pregnancy-related hypertension. We aim to answer the question if the functions of primary enzymes involved in cortisol metabolism: 11β-HSD1 and 11β-HSD2 and 5-reductases (both 5α- and 5β) are altered in the course of hypertensive pregnancy.

**Methods:**

We determined plasma and urinary cortisol and cortisone as well as their urinary tetrahydro- and allo-tetrahydrometabolites, both in free and conjugated forms in samples obtained from 181 Polish women in the third trimester of pregnancy. We compared steroid profiles in women with preeclampsia (PE), gestational hypertension (GH), chronic hypertension (CH) and in normotensives (controls).

**Results:**

We found significant differences in glucocorticoid balance in pregnancy-related hypertension. Plasma cortisol to cortisone was significantly lower in PE than in controls (3.00 vs. 4.79; *p* < 0.001). Increased function of renal 11β-HSD2 in PE and GH was manifested by significantly lower urinary free cortisol to cortisone ratio (0.169 and 0.206 vs. 0.277 in controls; *p* < 0.005). Markedly enhanced metabolism of cortisol was observed in pregnancy-related hypertension, with no significant alterations in CH, and the changes were more clearly expressed in PE than in GH.

**Conclusions:**

The glucocorticoid balance in PE and GH is shifted towards decreasing cortisol concentration either due to intensified conversion to cortisone or enhanced production of tetrahydro and allo-tetrahydrometabolites.

## Introduction

Pathogenesis of preeclampsia (PE) is not fully explained. It is believed to be initiated by the placental ischemia. In PE, the invasion of the trophoblast is aberrant; the spiral arteries remodeling is impaired. Therefore they are narrowed and resistive. Placental hypoperfusion generates oxidative as well as endoplasmic reticulum stress, and the release of antiangiogenic factors into the circulation (soluble fms-like tyrosine kinase—sFlt-1, and soluble endoglin—sEng) [1, 2]. The imbalance between pro and antiangiogenic factors leads to endothelial dysfunction [1, 3]. Oxidative stress also induces the release of proinflammatory cytokines and chemokines [2]. The systemic endothelial dysfunction, occurring in PE, is manifested in glomerular endotheliosis and proteinuria, hypertension, as well as coagulation abnormalities [3].

A healthy pregnancy is a state of hyperactivity of HPA axis and of hypercortisolism [4, 5]. Elevated cortisol (F) levels can be partially attributed to the estrogen-stimulated increase of corticosteroid-binding globulin (CBG). In normal pregnancy CBG levels rise with advancing gestation, with a corresponding increase of total plasma F, with a decline from 36 weeks onwards; this decline results in the rise of free F in maternal plasma [5]. On the other hand, the placenta is an important source of CRH, which further stimulates the release of maternal ACTH. It leads to the elevation of F levels in the course of pregnancy [4, 5]. F influences the hypothalamic CRH in a negative feedback loop, while the placental CRH is strongly stimulated by F in a mechanism of positive feedback loop [5]. During normal labor, maternal CRH, ACTH and F levels increase in maternal plasma and then drop at 4 days postpartum [4].

Lately, more and more is discussed the improper metabolism of glucocorticoids (GCs) in pregnancies with adverse outcomes [6, 7]. F is metabolized mainly by the 11β-hydroxysteroid dehydrogenase (11β-HSD), of which two isoforms (type 1 and type 2) have been described. 11β-HSD2 inactivates F to cortisone (E) and is localized mainly in the kidneys and the placenta, while 11β-HSD1 regenerates E to F in the liver and the fat tissue. Both F and E are then catabolized by 5α- and 5β-reductase in the liver and converted into tetrahydro- and allo-tetrahydrometabolites [8, 9].The disturbed activity of placental 11β-HSD2 in the course of PE was emphasized in last years [6]. It has not been yet discovered whether the abnormal function of 11β-HSD2 occurs in PE only locally (in the placenta) or it affects the whole body of a pregnant patient. Most research groups focus solely on the placental 11β-HSD2 as the enzyme localized in the tissue, which directly influences the proper development of the fetus [7]. Few available studies concerning the function of non-placental 11β-HSD2 in GH and PE are still inconclusive [10–12].

There are limited data reporting levels of F and E metabolites in pregnant women [13–15], some of which refers to spot urine only [14]. Others include women in the first weeks of pregnancy [15] or—like the study from 1980—concern only the tetrahydrometabolites of F and E assessed in samples from 6 patients [16]. To our best knowledge, the only analysis of the function of 11β-HSD1 in GH and PE was published more than 20 years ago and comprised small study population [10]. However, the recent data showed that some polymorphisms in *HSD11B1* gene, encoding 11β-HSD1, are linked to higher risk of developing PE [17]. Moreover, some authors revealed the increased levels of 11β-HSD1 in decidua of PE women [18]. These facts show the need for further research in the field of 11β-HSD function in pregnancy.

The aim of this study was to evaluate the overall maternal GC balance in pregnancy-related hypertension (both GH and PE) and to verify if the functions of primary enzymes involved in F metabolism: 11β-HSD1, 11β-HSD2, 5α and 5β-reductase are altered in hypertensive pregnancy. Plasma and urinary F and E, as well as their metabolites in urine, were determined and served as a basis for further indirect assessment of enzymes functions. The research question was whether the F conversion is impaired in the body of women suffering from GH and/or PE.

## Materials and methods

### Study participants

The study included 181 Polish women in the third trimester of pregnancy (at least 27th week of gestation, WG), recruited between 2013 and 2016 from inpatients of the Gynecological and Obstetric Clinical Hospital as well as healthy volunteers attending antenatal classes in Poznan.

The pregnant women were divided into four groups (Table 1). The hypertensive population comprised patients with chronic hypertension (CH) and pregnancy-related hypertension divided into GH and PE. CH patients were included as a separate group to verify if the observed changes are specific for pregnancy, and not due to hypertension per se. The normotensive (<140/90 mm Hg, according to WHO [19]) pregnant women served as a control group. CH was diagnosed in women who suffered from hypertension before pregnancy or who developed it before 20th WG. GH was diagnosed in pre-pregnancy normotensive women with de novo hypertension after 20th WG, without significant proteinuria. PE was diagnosed in patients with hypertension (pre-existing or developing in pregnancy) and the new onset of substantial proteinuria after 20th WG (protein excretion > 0.3 g/24 h) [20]. The hypertensive patients were treated with the antihypertensive drugs that are recommended to use during pregnancy. The hypothyroid patients were treated with levothyroxine; two asthmatic women were taking inhaled GCs (budesonide).

**Table 1:** Characteristics of the patients

	Controls	PE	GH	CH
Characteristics*	(*n* = 63)	(*n* = 42)	(*n* = 47)	(*n* = 29)
Age (y)^a^	30.4 ± 4.7	32.0 ± 5.1	30.2 ± 4.3	31.3 ± 6.8
• Age ≥ 35^b^	11 (17.5%)	13 (31.0%)	7 (14.9%)	9 (31.0%)
BMI before pregnancy (kg/m^2^)^c^	21.4 (19.6–23.8)	24.1 (21.7–26.0)^2^	25.0 (22.6–29.4)^3^	25.2 (20.6–32.0)^1^
• BMI ≤ 18.5^b^	6 (9.5%)	1 (2.5%)	0 (0.0%) ^1^	3 (10.3%)
• BMI ≥ 25^b^	12 (19.0%)	13 (32.5%)	23 (50.0%)^3^	15 (51.7%)^2^
GA at sample collection (wks)^c^	37 (33–39)	34 (31–37)^1^	37 (34–38)	35 (32–37)
Labour information				
• Nulliparity^b^	26 (41.3%)	28 (66.7%)^1^	25 (53.2%)	17 (58.6%)
• GA at delivery (wks)^b^	39 (38–40)	36 (33–38)^3^	39 (37–39)	39 (38–39)
• Prematurity^b^	4 (6.8%)	22 (55.0%)^3^	6 (12.8%)	2 (7.1%)
• Caesarean section^b^	35 (59.3%)	31 (81.6%)	26 (57.8%)	19 (73.1%)
• Infant birth weight (g)^c^	3490 (3100–3800)	2030 (1465–3020)^3^	3120 (2680–3600)^1^	3430 (2940–3720)
• SGA^b^	0 (0.0%)	19 (48.7%)^3^	12 (25.5%)^3^	5 (18.5%)^2^
• Female fetus^b^	23 (39.0%)	19 (48.7%)	24 (51.1%)	13 (46.4%)
Medical history				
• Hypothyroidism^b^	7 (11.1%)	7 (16.7%)	10 (21.3%)	3 (10.3%)
• Gestational diabetes^b^	5 (7.9%)	6 (14.3%)	6 (12.8%)	5 (17.2%)
• Asthma^b^	0 (0.0%)	1 (2.4%)	3 (6.4%)	2 (6.9%)
Medical therapy				
• Methyldopa^b^	0 (0.0%)	38 (95.0%)^3^	41 (100.0%)^3^	25 (86.2%)^3^
• Metoprolol^b^	2 (3.3%)	6 (15.0%)^1^	3 (6.4%)	4 (13.8%)
• Nitrendypine^b^	1 (1.6%)	20 (50.0%)^3^	4 (8.5%)	6 (20.7%)^2^
• verapamil^b^	6 (9.8%)	3 (7.3%)	6 (12.8%)	2 (6.9%)
• Magnesium sulfate^b^	0 (0.0%)	11 (27.5%)^3^	2 (4.3)	0 (0.0%)

*PE* pre-eclampsia, *GH* gestational hypertension, *CH* chronic hypertension

*cases with missing information are excluded

^1^*p* < 0.05; ^2^*p* < 0.005; ^3^*p* < 0.01

^a^values presented as mean ± SD

^b^values given as *n* (%)

^c^values shown as median (interquartile range)

Pre-pregnancy BMI was based on the maternal self-report at the enrollment. Prematurity was recognized when delivery occurred before 37 WG. Small for gestational age (SGA) babies were defined as those whose birth weight was below the 10th percentile for gestational age (GA) and gender, according to data for Wielkopolska region [21]. Giving birth to the SGA child was an exclusion criterion for controls. The exclusion criteria for the whole study population were: multiple pregnancy, pre-pregnancy diabetes, other than hypothyroidism endocrine diseases, mental and liver disorders (including cholestasis which was associated with reduced placental 11β-HSD2 expression [22]), infectious diseases, and stillbirth.

The study protocol was in accordance with the Helsinki Declaration and written informed consent was received from each participant. Ethical Committee at Poznan University of Medical Sciences approved the protocol.

### Determination of steroids in plasma and urine

Each woman was required to provide one urine sample from the 24h-urine collection and one morning blood sample as described in detail previously [12]. Finally, plasma samples were obtained from 170 while urine samples from 177 participants.

Total plasma F and E were determined by a validated HPLC-FLD method [12]. The amounts of urinary free and total steroids were assessed applying a validated HPLC-MS/MS method [23]. Six urinary GCs were determined: F, E, and their metabolites (tetrahydrocortisol, THF; tetrahydrocortisone, THE; allo-tetrahydrocortisol, alloTHF; allo-tetrahydrocortisone, alloTHE). Urinary free F and E were described as UFF and UFE, while free metabolites of F and E as THF_free_, THE_free_, alloTHF_free_, alloTHE_free_. The total urinary steroids, obtained after hydrolysis with Helix pomatia, were marked with the subscript “tot”: F_tot_, E_tot_, THF_tot_, THE_tot_, alloTHF_tot_, alloTHE_tot_. The amounts of steroids excreted per day were evaluated based on the 24-h urine volume and were expressed as µg of steroid compound per mmol of urinary creatinine (Cr). Each time when the sum of THF and alloTHF was used in calculations they were described as “THFs”, and consequently the sum of THE and alloTHE—as “THEs”.

### Parameters reflecting the GC balance

The functions of 11β-HSD, 5α and 5β-reductase were thoroughly estimated using the recognized parameters [8, 9, 24–28]. In contrast to other authors who do not consider alloTHE, we used both THE and alloTHE for accurate calculations, similarly to THF and alloTHF. As in the published literature there is no consistency which forms of compounds (free [8, 27] or total [9, 28]) should be used in most ratios, we calculated the ratios twice (using unconjugated and conjugated steroids) to compare the results.

The following parameters were defined:

1. for 11β-HSD2—UFF/UFE, considered as the best predictor of the function of 11β-HSD2
2. for 11β-HSD1—the F and E metabolites ratios: THFs_tot_/THEs_tot_ and THFs_free_/THEs_free_, providing an index of global 11β-HSD activity; elevated values of the ratios (with the normal UFF/UFE values) suggest an abnormal function of 11β-HSD1
3. for 5α/β-reductase—alloTHF_tot_/F_tot_ and THF_tot_/F_tot_ reflecting the activity of 5α and 5β-reductase, respectively; alloTHF_tot_/THF_tot_, assessing the overall balance between 5α and 5β-reductase and indicating which pathway of F metabolism dominates: the reduction to alloTHF or to THF
4. for net glucocorticoid balance in the body: plasma F/E; urinary F_tot_/E_tot_; THFs_tot_/UFF, which shows the irreversible conversion of F to its metabolites. (THFs_tot_ + THEs_tot_)/UFF represents a non-invasive measure of the metabolic clearance of F. (THFs_tot_ + THEs_tot_)/(UFF + UFE) represents a more complex index of total 11β-HSD activity. The sum of four major urinary GC metabolites (THFs_tot_ + THEs_tot_, normalized for urinary Cr) indicates the GC secretion.

Additionally, the levels of conjugation with glucuronic and sulphuric acid were evaluated for urinary F, E, THF, and THE. Those levels were calculated for each steroid as the ratio of free and total compound excreted per 24 h and were expressed in percentages (%).

### Statistical analysis

The study was performed in a case-control structure. The statistical analysis was accomplished using Statistica 12.0 software (StatSoft Inc., Tulsa, OK, USA). In each analysis, a *p*-value of <0.05 was considered significant. Prior to analysis, the data were checked for normal distribution with the Shapiro-Wilk test. For continuous variables, the differences between non-parametric data were estimated using the Mann–Whitney *U* test while in other cases, the analysis of variance (ANOVA test) was applied. The differences in categorical data were tested with Fisher’s exact test. The normally distributed data were reported as a mean ± standard deviation, the non-normally distributed data as medians (interquartile range) and the categorical data as a number of patients (%). The Spearman test was performed in each group to check for simple correlations between the calculated parameters and GA.

Multiple regression analysis was used to detail the results from Mann–Whitney *U* test. The results were reported only when the built models were significant (p-value was <0.05). In all analyses based on multiple regression, the cases with residuals (the differences between the observations and the estimated values) greater than three standard deviations were not included in the analysis.

## Results

### Plasma and urinary steroids

The plasma and urinary GCs levels observed in the study groups (Mann–Whitney *U* test) are presented in Supplementary Table 1.

#### Results below the lower limit of quantification (LLOQ) or the limit of detection (LOD)

During analyses, a few patients (mostly from PE group) presented results of free steroids < LLOQ or even <LOD. In one case (GH group) the level of alloTHF_tot_ was <LOD. Evidently, the results < LLOQ are measured with lower precision and accuracy and the values < LOD are impossible to estimate. They are, however, still a valuable source of information. Therefore, omitting them in further calculations could overestimate the results obtained by the study group and even lead to the false conclusions [29]. Considering above, we decided not to exclude the patients with results < LLOQ or <LOD from the statistics. We applied the following approaches to minimize the potential error:

- All detectable concentrations were analyzed statistically (also the results between LLOQ and LOD), as shown previously by others in pharmacokinetics studies [29]. The method was applied to plasma E in four patients. The results < LLOQ were also obtained for urinary alloTHF_free_ and alloTHE_free_ in >97% of samples. Therefore, obviously, the results for alloTHF_free_ and alloTHE_free_ were not shown Supplementary Table 1, and they were excluded from the further analyses. The THFs_free_ and THEs_free_ were, therefore, equal to THF_free_ and THE_free_, respectively.
- For undetectable steroids (the results < LOD), the concentrations were calculated by dividing the LOD by the square root of 2, as recommended in the literature for studies when relatively few data are <LOD [30]. This approach is used in clinical studies considering patients with enzyme deficiency [31]. The method was applied to replace the missing data for UFF in four patients, THF_free_ in four patients and alloTHF_tot_ in one patient. The censored concentrations for UFF were replaced by 0.28 ng/mL (LOD = 0.4 ng/mL), while for THF_free_ and alloTHF_tot_ with 0.7 ng/mL (LOD = 1.0 ng/mL). Such obtained results were then multiplied by the urine volume (and normalized for Cr level when necessary) and used for further calculations.

#### Results higher than the ULOQ

The THE_tot_ concentrations for 8 patients were >ULOQ (5000 ng/mL in the matrix). For those patients, the urine sample was diluted and re-assessed (the dilution integrity was estimated during method validation [23]).

### Parameters reflecting the GC balance

The values of parameters indicating GC secretion and assessing the function of 11β-HSD2, 11β-HSD1, 5α, and 5β-reductase, as well as the overall balance between those enzymes, are presented in Table 2. The table also contains the results from Mann–Whitney *U* test.

**Table 2:** Parameters reflecting function of 11β-HSD1, 11β-HSD2, 5α, and 5β-reductases as well as the overall glucocorticoid balance in the body

	Controls	PE	GH	CH
*Function of 11β-HSD2*				
UFF/UFE	0.277 (0.198–0.373)	0.169^3^ (0.115–0.221)	0.206^2^ (0.158–0.261)	0.282 (0.207–0.421)
*Function of 11β-HSD1*				
THFs_tot_/THEs_tot_	0.280 (0.235–0.337)	0.346^1^ (0.256–0.445)	0.318 (0.247–0.373)	0.271 (0.228–0.385)
THF_free_/THE_free_	0.498 (0.353–0.720)	0.416* (0.268–0.601)	0.476 (0.347–0.606)	0.428 (0.264–0.597)
*Function of 5α and 5β-reductases*				
alloTHF_tot_/F_tot_	0.0477 (0.0248–0.0805)	0.0661 (0.0264–0.1354)	0.0489 (0.0282–0.0916)	0.0391* (0.0178–0.0487)
THF_tot_/F_tot_	2.20 (1.63–3.38)	3.51^3^ (2.15–6.11)	3.22^2^ (2.25–4.59)	3.19* (1.85–4.48)
alloTHF_tot_/THF_tot_	0.0203 (0.0133–0.0329)	0.0179 (0.0096–0.0249)	0.0151* (0.0070–0.0330)	0.0106^2^ (0.0074–0.0183)
*Overall glucocorticoid balance in the body*				
Plasma F/E	4.79 (3.66–5.76)	3.00^3^ (2.57–4.21)	4.01* (3.53–5.26)	4.94 (3.44–6.07)
Urinary F_tot_/E_tot_	1.14 (0.86–1.30)	1.08 (0.86–1.31)	1.04 (0.92–1.26)	1.11 (0.89–1.43)
THFs_tot_ + THEs_tot [_µg/mmol Cr]	380.1 (292.4–504.5)	394.2 (253.8–659.7)	489.7^1^ (346.6–699.3)	450.3 (302.8–623.5)
THFs_tot_/UFF	16.80 (11.50–25.64)	36.95^3^ (23.04–85.05)	33.07^3^ (18.62–45.22)	23.78* (13.96–37.59)
(THFs_tot_ + THEs_tot_)/UFF	76.39 (52.28–117.21)	140.63^3^ (91.93–322.45)	138.19^3^ (81.07–178.36)	109.81* (66.52–155.77)
(THFs_tot_ + THEs_tot_)/(UFF + UFE)	15.42 (11.39–23.51)	19.47^1^ (13.95–35.80)	23.58^1^ (14.67–33.12)	22.58 (12.52–35.30)
*Steroid conjugation with glucuronides and sulphates*				
F conjugation degree [%]	85.0 (81.2–89.5)	90.9^3^ (87.1–94.2)	88.9^1^ (85.2–91.4)	87.1 (76.3–91.6)
E conjugation degree [%]	44.2 (31.3–53.8)	42.6 (33.7–47.9)	42.3 (31.2–49.1)	39.0 (30.2–52.1)
THF conjugation degree [%]	98.8 (98.3–99.0)	99.0^1^ (98.6–99.2)	99.0^1^ (98.6–99.2)	98.8 (98.6–99.0)
THE conjugation degree [%]	99.3 (99.0–99.5)	99.2 (98.8–99.4)	99.3 (99.0–99.5)	99.2 (98.4–99.4)

Results are presented as medians (interquartile range) for groups of women with preeclampsia (PE), gestational hypertension (GH), chronic hypertension (CH) and normotensive controls

*close to be significant (*p* < 0.09)

^1^*p* < 0.05; ^2^*p* < 0.005; ^3^*p* < 0.001

Multiple regression analysis was applied to particularize the Mann–Whitney *U* test. The PE group was characterized by significantly earlier GA at sample collection as compared to controls. Therefore, GA at sample collection was considered as an unintended confounder, and the models containing each parameter (as the dependent variable), PE and GA at sampling (as independent variables) were built. The results (Supplementary Table 2) confirmed that the dependences observed in Mann–Whitney *U* test are the consequence of the disease and not the earlier sampling time.

Furthermore, the stepwise forward multiple regression was performed (selection *F* = 1, elimination *F* = 0). The dependent variables in such models were the parameters calculated from endogenous GC levels. The independent variables included in all models were: a hypertensive disorder of pregnancy (respectively PE, GH or CH) along with the factors that may influence GC equilibrium (maternal age, hypothyroidism, diabetes, baby’s gender, nulliparity, GA at sampling and pre-pregnancy BMI). The most important results from the stepwise multiple regression analyses reporting the associations between parameters and particular disease (PE, GH or CH) are presented in Table 3. The details are shown in Supplementary Table 3. Each time, a semi-partial correlation (R) and a p-value were noted.

**Table 3:** Results of the forward multiple regression analyses assessing the relationship between calculated parameters and hypertensive disorder of pregnancy (PE, GH or CH, each in the separate model) after adjustment for factors potentially influencing F metabolism

	PE	GH	CH
UFF/UFE	*R* **=** **−0.516;** *p* < **0.001**	R = **−**0.271; *p* = 0.004	NS
THFs_tot_/THEs_tot_	*R* = 0.198; *p* = 0.045	NS	NS
THF_free_/THE_free_	*R* = **−0.214;** *p* = **0.034**	NS	NS
alloTHF_tot_/F_tot_	*R* = 0.338; *p* < 0.001	NS	NS
THF_tot_/F_tot_	*R* = 0.424; *p* < 0.001	*R* = 0.233; *p* = 0.013	NS
alloTHF_tot_/THF_tot_	NS	NS	*R* = **−0.252;** *p* = **0.028**
plasma F/E	*R* = **−0.473;** *p* < **0.001**	NS	NS
urinary F_tot_/E_tot_	NS	NS	NS
THFs_tot_ + THEs_tot_ [µg/mmol Cr]	NS	*R* = **0.265;** *p* = **0.007**	NS
THFs_tot_/UFF	*R* = 0.424; *p* < 0.001	*R* = **0.430;** *p* < **0.001**	NS
(THFs_tot_ + THEs_tot_)/UFF	*R* = 0.484; *p* < 0.001	*R* = **0.342;** *p* < **0.001**	NS
(THFs_tot_ + THEs_tot_)/(UFF + UFE)	*R* = 0.342; *p* < 0.001	*R* = **0.206;** *p* = **0.029**	NS
F conjugation degree [%]	*R* = 0.467; *p* < 0.001	*R* = 0.236; *p* = 0.010	NS
E conjugation degree [%]	NS	NS	NS
THF conjugation degree [%]	*R* = **0.242;** *p* = **0.019**	*R* = **0.246;** *p* = **0.013**	NS
THE conjugation degree [%]	*R* = **−**0.233; *p* = 0.019	NS	NS

Each time, a semi-partial correlation (*R*) and a *p*-value were noted. The associations where the sole predictor of the response variable was the hypertensive disorder of pregnancy are marked in bold

*UFF* urinary free cortisol, *UFE* urinary free cortisone, *THF* tetrahydrocortisol, *THE* tetrahydrocortisone, *alloTHF* allo-tetrahydrocortisol, *alloTHE* allo-tetrahydrocortisone, *NS* not significant

The stepwise multiple regression revealed that PE significantly influenced most of the analyzed parameters. In most cases, PE was the sole significant predictor of the response variables. The analysis showed that GH was the single significant predictor of few response variables. The CH was the sole significant predictor of alloTHF_tot_/THF_tot_—the tendency seen in Mann–Whitney *U* test was confirmed.

### Conjugation level of particular steroids

The degrees of conjugation with glucuronic and sulphuric acid for certain steroids are presented in Table 2. The Spearman tests showed that in normotensive pregnant women, the conjugation degree of F and E is increasing with GA (*R* = 0.485; *p* < 0.0001 for F and *R* = 0.264; *p* = 0.038 for E). Such observations were not confirmed in a hypertensive population. Conversely to controls, in PE group there was a negative correlation between conjugation degree of E and GA (*R* = −0.342; *p* = 0.027) and a similar tendency in GH (*R* = −0.293; *p* = 0.051). In CH patients, we observed only the trend between conjugation of F and GA (*R* = 0.375; *p* = 0.059).

The stepwise multiple regression analyses were performed, similarly to those described in results section 3.2. The results reporting the associations between GC conjugation degree and particular disease (PE, GH or CH) are presented in Table 3. The detailed results, also showing the influence of other cofactors, are presented in Supplementary Table 3.

## Discussion

The disturbed GC balance was reported in many medical conditions, inter alia the polycystic ovary syndrome [32, 33], metabolic syndrome, insulin resistance [25], cholestasis [34] and mental disorders [27]. They are usually associated with the abnormal activity of 11β-HSD, 5α and/or 5β-reductase. Here we present that the disturbed metabolism of F is also manifested in women suffering from pregnancy-related hypertension, including both PE and GH.

Our preliminary study [12] surprisingly revealed that, despite the literature data proving the diminished expression and function of placental 11β-HSD2 in PE [35–38], the apparent activity of renal 11β-HSD2 is significantly increased in that condition. This finding was in contradiction to other results. Heilmann et al. [11] reported higher UFF/UFE ratio in the group comprising GH and PE patients indicating decreased function of 11β-HSD2. Walker et al. [10] presented no differences in UFF/UFE ratio between hypertensive pregnant women and controls. Our present study, conducted on a larger population and extended by the analysis of urinary metabolites of F and E, entirely confirms our previous observations. Patients with PE presented significantly lower values of UFF/UFE than controls, and this difference remained significant after adjustment for confounders (Table 3). One need to emphasize that this paper presents the results obtained using HPLC-MS/MS method [23] instead of HPLC-FLD [12], and the mass spectrometry is known as the most specific and accurate way of detection. However, independently of the method applied, we observed the same dependencies in urinary F and E. We suggest that the diminished function of placental 11β-HSD2 in PE, reported by others, might be a kind of compensatory mechanism limiting maternal F levels that reach the fetus. The reduced UFF/UFE ratio could be attributed to the substantial proteinuria in PE patients as some authors reported increased function of renal 11β-HSD2 in non-pregnant proteinuric patients [39]. However, significantly lower UFF/UFE ratio also in usually non-proteinuric GH women suggests that other mechanisms should be considered to explain the enhanced 11β-HSD2 function in pregnancy-related hypertension. At the same time, CH group presented similar UFF/UFE ratios as controls, what confirms our previous conclusions [12] that the enhanced function of renal 11β-HSD2 concerns only women with pregnancy-related hypertension (PE and GH).

We found that PE is associated with higher THFs_tot_/THEs_tot_ ratio (Table 2) what might indicate the increased function of 11β-HSD1 in that condition. On the one hand, the THFs_tot_/THE_tot_ is a non-specific parameter reflecting more precisely the overall balance between both 11β-HSD isoforms [9, 13, 39] than the actual function of 11β-HSD1. It is widely used in the clinical practice in the diagnosis of apparent mineralocorticoid excess, as lack or diminished function of 11β-HSD2 implies much higher UFF/UFE ratio and also significantly increased THFs/THE [24, 40]. The literature data, however, show that in case of the proper function of 11β-HSD2 (reflected in normal value of UFF/UFE), the THFs/THE ratio describes quite acceptably the function of 11β-HSD1 [8, 28]. Our PE patients obtained not lower (as it could be supposed based on the lower UFF/UFE values) but higher THFs_tot_/THEs_tot_ ratio as compared to controls (0.346 vs. 0.280). When analyzing not total but free metabolites of F and E (THF_free_/THE_free_ ratio), one will get similar conclusions to those derived from UFF/UFE, as PE was associated with lower THF_free_/THE_free_ (R = −0.214; *p* = 0.034). Considering UFF/UFE, THF_free_/THE_free_ and THFs_tot_/THEs_tot_ we conclude that they reflect rather the augmented function of 11β-HSD2 than 11β-HSD1. The observed enhanced conjugation of F and THF in PE may further indicate the pursuit of woman’s body to the efficient removal of active GC and its major metabolite.

All mentioned observations may lead to the conclusion that the F metabolism is significantly intensified in PE. Firstly, the similar THFs_tot_ + THEs_tot_ indicates the comparable GC secretion (the equivalent activity of adrenal glands) in normotensive and preeclamptic pregnancy (380.1 vs. 394.2 µg/mmol Cr, respectively). However, plasma F concentration is markedly lower in PE (663 vs. 782 nmol/L), partially because of earlier GA at sampling, but also because of PE itself (Supplementary Table 3). Lower plasma F in PE suggests stronger GC metabolism in this group. The assumption of the enhanced F clearance could be strengthened by lower UFF/UFE in PE (increased 11β-HSD2 function) and significantly higher THF_tot_/F_tot_ and alloTHF_tot_/F_tot_ (increased functions of 5β- and 5α-reductase, respectively) (Table 3). Lastly, the parameter indicating the metabolic clearance of F: (THFs_tot_ + THEs_tot_)/UFF is almost two times higher in PE when compared to controls (141.59 vs. 76.95). Notably, lower plasma F in PE with comparable GC secretion (reflected in the amounts of tetrahydrometabolites in urine) may suggest the blunted HPA response in this condition. Lower F production in PE was previously reported by Ho et al. [5], who observed lower total and free F in maternal plasma in PE. They suggested it could result from the underactivity of maternal HPA axis.

Such conclusion about diminished HPA response cannot be derived from the results for GH group. Plasma F in GH patients was similar to the values obtained for normotensive women (Supplementary Table 1), in spite of evidently enhanced F metabolism. Intensified F metabolism is manifested in significantly lower UFF/UFE values (increased 11β-HSD2 function), higher THF_tot_/F_tot_ (increased 5β-reductase function) and almost two times higher (THFs_tot_ + THEs_tot_)/UFF (strongly enhanced F clearance). Moreover, the GC secretion is substantially increased. All these facts lead to the conclusion that the enhanced metabolism of F in GH patients is compensated by higher F secretion due to HPA activity (Table 2). Our observations do not corroborate with the results of Walker et al. [10] who showed no differences in 11β-HSD function between PE, GH and normotensive pregnant women. Such discrepancies may result from relatively small study population in the cited study (13 controls, 7 women with GH and 8 with PE). Moreover, it is worth noting that we calculated F and E metabolites ratio considering not only THF and THE but also allo-tetrahydrometabolites.

The results obtained for patients with CH are very similar to those for normotensive women. However, worth noting is the fact that the balance in functions of 5α and 5β-reductases is shifted towards 5β-reductase (lower alloTHF_tot_/THF_tot_ values in CH, as presented in Tables 2 and 3). Surprisingly, this is in contradiction to results for non-pregnant hypertensive population presenting decreased activity of 5β-reductase [8].

We are aware of limitations of our study. During the study, in 2016, the diagnostic criteria of PE changed [41], and proteinuria is no longer obligatory for the diagnosis. We maintained the criteria with substantial proteinuria to ensure similar ones for all recruited patients, and for better comparability with previously published results. An important limitation is also the significantly earlier GA at sampling in PE group as compared to controls. Our study population comprised women who were admitted to hospital for routine tests or due to abnormalities arising during the course of pregnancy. Patients with PE, as a result of their rapidly deteriorating condition, were then usually enrolled to the hospital much earlier than other pregnant women. Secondly, the groups were poorly matched in regard to pre-pregnancy BMI. Moreover, women with PE were significantly more often nulliparous. The tendency to higher-pregnancy BMI among PE and GH patients, as well as the higher rates of nulliparity in PE but not in GH, were previously reported and these features are mentioned among risk factors of pregnancy-specific hypertension [42]. Obesity and higher BMI are also linked to hypertension in general non-pregnant population [43]. As those confounders could adversely affect the results, the multiple regression analyses were performed to confirm our observations.

Strengths of our research are the relatively large group of patients as compared to other published studies [10] and thorough analysis of GC balance in pregnant women. We assessed the function of primary enzymes involved in F metabolism: 11β-HSD1, 11β-HSD2, 5α and 5β-reductase and the overall equilibrium between them. Additionally, the study group included both PE and GH patients, what brings the new insight into phenomena associated with pregnancy-related hypertension.

Our main findings include: markedly intensified F metabolism manifested in the increased function of renal 11β-HSD2, 5α and 5β-reductase in PE as well as the enhanced function of renal 11β-HSD2 and 5β-reductase in GH. The GC balance in PE is clearly shifted towards decreasing F concentration either due to the intensified conversion of F to E or enhanced production of tetrahydro- and allo-tetrahydrometabolites. The observed changes are similar, however, less marked in GH. Importantly, we suggest the blunted response of HPA axis in PE, what was not found in other hypertensive disorders of pregnancy. Further studies concerning non-pregnant women with a history of PE and GH are needed to assess whether the observed changes in GC balance are limited to gestation, or they remain in the body of affected women after pregnancy. Moreover, the comprehensive prospective study, including women with high risk of PE and GH, could bring the answer if any interference in GC balance during hypertensive pregnancy would be justified.

## Electronic supplementary material

Supplementary Information
