# Supplementary material for: Increased cortisol metabolism in women with pregnancy-related hypertension
Source: Endocrine. 2018 Apr 2;61(1):125–33. doi: 10.1007/s12020-018-1586-4 (PMC5997110; doi:10.1007/s12020-018-1586-4)
Supplement: Supplementary file 1 — Supplementary Information [file 12020_2018_1586_MOESM1_ESM.docx]

Katarzyna KOSICKA^1^, Anna SIEMIĄTKOWSKA^1^, Agata SZPERA-GOŹDZIEWICZ^2^, Mariola KRZYŚCIN^2^, Grzegorz H. BRĘBOROWICZ^2^, Franciszek K. GŁÓWKA^1^

Increased cortisol metabolism in women with pregnancy-related hypertension

^1^ Department of Physical Pharmacy and Pharmacokinetics, Poznan University of Medical Sciences, 6 Święcickiego Street, 60-781 Poznań, Poland

^2^ Department of Perinatology and Gynecology, Poznan University of Medical Sciences, 33 Polna Street, 60-535 Poznań, Poland

ONLINE SUPPLEMENT

**Supplementary Table 1.** Plasma and urinary F and E levels as well as their urinary tetrahydro- and allo-tetrahydrometabolites in hypertensive and normotensive pregnant women. Statistically significant differences are marked in red.

|  | **controls** | **PE** | **GH** | **CH** |
| --- | --- | --- | --- | --- |
| total steroids in plasma | **(n = 54)** | **(n = 42)** | **(n = 46)** | **(n = 28)** |
| **F** [nmol/L] | 782.15  (654.41-943.00) | 662.99 ^1^  (451.63-811.89) | 721.45  (609.11-987.30) | 727.41  (587.37-988.96) |
| **E** [nmol/L] | 171.31  (132.39-201.27) | 191.60  (153.33-234.99) | 188.36  (148.65-214.17) | 157.46  (139.34-190.03) |
| free steroids in urine ^a^ | **(n = 63)** | **(n = 42)** | **(n = 46)** | **(n = 26)** |
| **UFF/Cr** [µg/mmol] | 5.60  (4.12-7.23) | 3.10 ^3^  (1.70-4.73) | 4.30 ^2^  (3.03-5.33) | 4.19  (2.83-7.34) |
| **UFE/Cr** [µg/mmol] | 18.11  (13.26-25.09) | 17.90  (11.76-24.61) | 18.39  (14.56-26.00) | 17.11  (11.90-23.69) |
| **THF_free_/Cr** [µg/mmol] | 1.13  (0.77-1.63) | 1.10  (0.52-1.60) | 1.18  (0.75-1.79) | 1.35  (0.82-1.92) |
| **THE_free_/Cr** [µg/mmol] | 2.08  (1.35-3.68) | 2.50  (1.23-3.76) | 2.53  (1.46-4.04) | 2.72 *  (1.61-5.56) |
| total steroids in urine ^a^ | **(n = 62)** | **(n = 42)** | **(n = 45)** | **(n = 26)** |
| **F_tot_/Cr** [µg/mmol] | 37.97  (30.21-48.23) | 32.28 ^1^  (21.33-38.7) | 35.50  (28.82-43.97) | 35.41  (28.18-39.31) |
| **E_tot_/Cr** [µg/mmol] | 35.52  (28.52-40.60) | 28.58 ^1^  (20.26-40.02) | 34.93  (28.18-40.85) | 28.24 ^1^  (25.61-35.68) |
| **alloTHF_tot_/Cr** [µg/mmol] | 1.90  (1.07-2.89) | 1.43  (0.85-3.48) | 1.90  (0.84-3.33) | 1.13 ^1^  (0.64-1.71) |
| **THF_tot_/Cr** [µg/mmol] | 84.02  (66.50-115.20) | 107.08  (53.12-142.17) | 120.66 ^2^  (84.32-163.80) | 99.63  (67.15-145.04) |
| **alloTHE_tot_/Cr** [µg/mmol] | 2.90  (1.90-5.43) | 2.41 *  (1.48-3.90) | 2.97  (2.04-3.92) | 2.57  (1.78-5.41) |
| **THE_tot_/Cr** [µg/mmol] | 295.79  (223.72-406.28) | 275.81  (194.46-510.06) | 377.64 ^1^  (259.70-537.00) | 349.09  (218.68-501.02) |
| **urine volume** [ml/24h] | 1530  (1100 - 1900) | 2125 ^1^  (1300 - 2750) | 1750  (1100 - 2350) | 1600  (1010 - 2240) |
| **urinary Cr** (mmol/24h) | 8.21  (6.81 - 10.15) | 9.27 ^1^  (8.04 - 11.51) | 9.42  (7.74 - 10.68) | 8.34  (6.41 - 11.26) |

^1^ p<0.05; ^2^ p<0.005; ^3^ p< 0.001; * close to be significant (p<0.09); ^a^ results for urinary steroids were each time normalized for urinary creatinine (Cr) level.

**Supplementary Table 2.** Results from the multiple regression analyses assessing the relationship between calculated parameters and pre-eclampsia (PE) after adjustment for gestational age (GA) at sampling. Each time, a semi-partial correlation (R) and a p-value were noted.

|  | **PE** | **GA** |
| --- | --- | --- |
| **UFF/UFE** | R=-0.493; p<0.001 | R=-0.175; p=0.047 |
| **THFs_tot_/THEs_tot_** | R=0.202; p=0.034 | R=-0.256; p=0.008 |
| **THF_free_/THE_free_** | R=-0.280; p=0.004 | NS |
| **alloTHF_tot_/F_tot_** | NS | R=-0.233; p=0.019 |
| **THF_tot_/F_tot_** | R=0.308; p<0.001 | R=-0.254; p=0.006 |
| **alloTHF_tot_/THF_tot_** | NS | NS |
| **plasma F/E** | R=-0.433; p<0.001 | NS |
| **urinary F_tot_/E_tot_** | NS | NS |
| **THFs_tot_+THEs_tot_** [µg/mmol Cr/day] | NS | NS |
| **THFs_tot_/UFF** | R=0.438; p<0.001 | NS |
| **(THFs_tot_+THEs_tot_)/UFF** | R=0.398; p<0.001 | NS |
| **(THFs_tot_+THEs_tot_)/(UFF+UFE)** | NS | NS |
| **F conjugation degree** [%] | R=0.406; p<0.001 | R=0.229; p=0.013 |
| **E conjugation degree** [%] | NS | NS |
| **THF conjugation degree** [%] | NS | NS |
| **THE conjugation degree** [%] | NS | NS |

UFF-urinary free cortisol, UFE – urinary free cortisone, THF – tetrahydrocortisol, THE – tetrahydrocortisone, alloTHF – allo-tetrahydrocortisol, alloTHE – allo-tetrahydrocortisone; NS - not significant.

**Supplementary Table 3.** Detailed results of the forward multiple regression analyses assessing the relationship between calculated parameters and hypertensive disorder of pregnancy (PE, GH or CH, each in the separate model) after adjustment for factors potentially influencing F metabolism. Each time, a semi-partial correlation (R) and a p-value were noted. The influence of a particular hypertensive disorder is marked in red.

|  | **PE** | **GH** | **CH** |
| --- | --- | --- | --- |
| **UFF/UFE** | **PE:** R=-0.516; p<0.001 | **GH:** R=-0.271; p=0.004  **GA:** R=-0.256; p=0.006 | NS |
| **THFs_tot_/THEs_tot_** | **PE:** R=0.198; p=0.045  **GA:** R=-0.282; p=0.005 | NS | NS |
| **THF_free_/THE_free_** | **PE:** R=-0.214; p=0.034 | NS | NS |
| **alloTHF_tot_/F_tot_** | **PE:** R=0.338; p<0.001  **baby’s gender:**  R=-0.196; p=0.040  **GA:** R=-0.226; 0.018  **pre-pregnancy BMI:**  R=-0.219; p=0.022  **nulliparity:**  R=-0.237; p=0.013 | NS | NS |
| **THF_tot_/F_tot_** | **PE:** R=0.424; p<0.001  **GA:** R=-0.296; p<0.001  **nulliparity:**  R=-0.254; p=0.004  **diabetes:**  R=0.177; p=0.040 | **GH:** R=0.233; p=0.013  **GA:** R=-0.189; p=0.042 | **GA:** R=-0.326; 0.002  **pre-pregnancy BMI:** R=0.260, p=0.013 |
| **alloTHF_tot_/THF_tot_** | NS | NS | **CH:** R=-0.252; p=0.028 |
| **plasma F/E** | **PE:** R=-0.473; p<0.001 | NS | **GA:** R=-0.250; p=0.029 |
| **urinary F_tot_/E_tot_** | NS | NS | **GA:** R=0.326; p=0.003 |
| **THFs_tot_+THEs_tot_** [µg/mmol Cr] | **pre-pregnancy BMI:** R=0.201; p=0.049 | **GH:** R=0.265; p=0.007 | NS |
| **THFs_tot_/UFF** | **PE:** R=0.424; p<0.001  **pre-pregnancy BMI:**  R=-0.237; p=0.012 | **GH:** R=0.430; p<0.001 | **baby’s gender:**  R=-0.215; p=0.043 |
| **(THFs_tot_+THEs_tot_)/UFF** | **PE:** R=0.484; p<0.001  **baby’s gender:**  R=-0.192, p=0.037  **pre-pregnancy BMI:**  R=-0.210, p=0.023 | **GH:** R=0.342; p<0.001 | NS |
| **(THFs_tot_+THEs_tot_)/(UFF+UFE)** | **PE:** R=0.342; p<0.001  **baby’s gender:**  R=-0.197, p=0.042 | **GH:** R=0.206; p=0.029 | **pre=pregnancy BMI:** R=0.315; p=0.004 |
| **F conjugation degree** [%] | **PE:** R=0.467; p<0.001  **GA:** R=0.266; p=0.003  **pre-pregnancy BMI:**  R=-0.205; p=0.020  **diabetes:** R=-0.173; p=0.049 | **GH:** R=0.236; p=0.010  **GA:** R=0.394; p<0.001 | **GA:**  R=0.438; p<0.001 |
| **E conjugation degree**  [%] | NS | NS | NS |
| **THF conjugation degree**  [%] | **PE:** R=0.242; p=0.019  **hypothyroidism:**  R=-0.242; p=0.015  **pre-pregnancy BMI:**  R=0.227; p=0.022 | **GH:** R=0.246; p=0.013 | NS |
| **THE conjugation degree** [%] | **PE:** R=-0.233; p=0.019 | NS | **pre-pregnancy BMI:**  R=-0.309; p=0.004 |

Factors influencing F metabolism, which were included as confounders: maternal age, hypothyroidism, diabetes, baby’s gender, nulliparity, GA at sampling, pre-pregnancy BMI.
